# Management of triple-negative breast cancer by natural compounds through different mechanistic pathways

**DOI:** 10.3389/fgene.2024.1440430

**Published:** 2024-07-26

**Authors:** Mohammed Kaleem, Mandar Thool, Nitin G. Dumore, Abdulrasheed O. Abdulrahman, Wasim Ahmad, Amal Almostadi, Mohammad Hassan Alhashmi, Mohammad Amjad Kamal, Shams Tabrez

**Affiliations:** ^1^ Department of Pharmacology, Dadasaheb Balpande College of Pharmacy, Rashtrasant Tukadoji Maharaj Nagpur University, Nagpur, Maharashtra, India; ^2^ Department of Pharmaceutics, Dadasaheb Balpande College of Pharmacy, Nagpur, Maharashtra, India; ^3^ Department of Biochemistry, Faculty of Science, King Abdulaziz University, Jeddah, Saudi Arabia; ^4^ Department of KuliyateTib, National Institute of Unani Medicine, Bengaluru, India; ^5^ King Fahd Medical Research Center, King Abdulaziz University, Jeddah, Saudi Arabia; ^6^ Department of Medical Laboratory Sciences, Faculty of Applied Medical Sciences, King Abdulaziz University, Jeddah, Saudi Arabia; ^7^ Center for High Altitude Medicine, Institutes for Systems Genetics, West China School of Nursing, Frontiers Science Center for Disease-Related Molecular Network, West China Hospital, Sichuan University, Chengdu, China; ^8^ Department of Pharmacy, Faculty of Health and Life Sciences, Daffodil International University, Birulia, Bangladesh; ^9^ Centre for Global Health Research, Saveetha Medical College and Hospital, Saveetha Institute of Medical and Technical Sciences, Chennai, Tamil Nadu, India; ^10^ Enzymoics, Hebersham, NSW, Australia; Novel Global Community Educational Foundation, Hebersham, NSW, Australia

**Keywords:** DNA methylation, genes, histone modification, natural drugs, TNBC, epigenetic mechanims

## Abstract

Triple-negative breast cancer (TNBC) is the most severe form of breast cancer, characterized by the loss of estrogen, progesterone, and human epidermal growth factor receptors. It is caused by various genetic and epigenetic factors, resulting in poor prognosis. Epigenetic changes, such as DNA methylation and histone modification, are the leading mechanisms responsible for TNBC progression and metastasis. This review comprehensively covers the various subtypes of TNBC and their epigenetic causes. In addition, the genetic association of TNBC with all significant genes and signaling pathways linked to the progression of this form of cancer has been enlisted. Furthermore, the possible uses of natural compounds through different mechanistic pathways have also been discussed in detail for the successful management of TNBC.

## Graphical Abstract

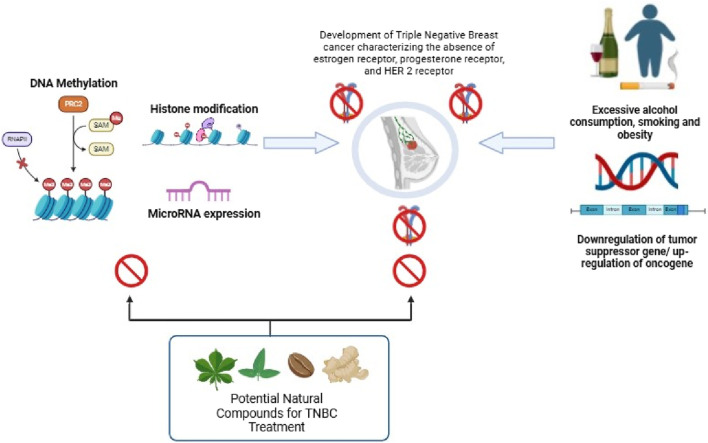

## 1 Introduction

Cancer is characterized by an uncontrolled growth of the body’s cells (Kaleem et al., 2022; Chunarkar-patil et al., 2024). Several genetic and epigenetic factors have been suggested to be responsible for the development of various types of cancer, resulting in the switch-off/on the tumor suppressor genes. Epigenetics involves studying the mechanisms that can change gene expression without affecting DNA sequence (Füllgrabe et al., 2011; Reddy Dachani et al., 2024). Several epigenetic modifications, such as histone acetylation and DNA methylation, are responsible for cancer development (Kaleem et al., 2021a; Kaleem et al., 2021b).

Breast cancer (BC) is the most common type of cancer among women globally (Michniak-Kohn et al., 2022; Kaleem et al., 2024). It is a highly complex form of cancer with distinct biological subtypes and several well-known prognostic markers of therapeutic importance (Hobbs et al., 2022). As per the report of 2020, nearly 2.3 million (11.7%) new cancer cases were diagnosed, and 6,84,996 (6.9%) deaths occurred due to breast cancer (Kaleem et al., 2022), and the incidence was anticipated to increase about 3 million new cases and one million mortalities by 2040 (Arnold et al., 2022). Several factors, such as sex, family history, genetic mutations, hormonal imbalance, diethylstilbestrol intake, obesity, alcohol consumption, smoking, vitamin D deficiency, radiation exposure, processed meat intake, etc., can cause breast cancer (Almansour, 2022). However, the prognosis and clinical manifestations vary considerably amongst patients. Notably, BC consists of human epidermal growth factor receptor 2 (HER2) overexpression, luminal A, luminal B, and subtypes of triple-negative breast cancer (TNBC) (Zhu et al., 2022).

TNBC is characterized by the loss of the cell surface’s progesterone, estrogen, and HER2 receptors. Among breast cancer, the incidence of TNBC is approximately 15%–20% and is more commonly reported in patients below 40 years (Bilani et al., 2020; Łukasiewicz et al., 2021). It has been noted to have higher death rates than other subcategories of BC. There is a significant decline in the lifespan of TNBC patients, approximately 45% develop metastatic tumors in the brain or other parts of the body (Schmid et al., 2018). Moreover, around 25% recovery percentage in TNBC patients has been reported by numerous studies (Zhu et al., 2022).

Although the scientific community has put significant efforts into discovering/developing potential moieties that can effectively mitigate breast cancer cell proliferation, their therapeutic efficacy is still very low (Hsu et al., 2022). The main reason is poor diagnosis since genomic changes in different cancers change their type, requiring specific treatments (Li Y. Z. et al., 2022). TNBC is a highly aggressive form of breast cancer, often results in the development of drug resistance via the expression of different efflux proteins on the cell surface, which decreases the treatment efficiency (Nedeljković and Damjanović, 2019). Along with the genetic changes, DNA methylation and histone modifications are epigenetic alterations that result in rapid development and metastasis of TNBC (Manoochehri et al., 2021). This article provide a detailed description of the genes linked to TNBC progression, their epigenetic modification, and the mechanism of action of natural compounds through the epigenetic modulation for TNBC treatment.

## 2 Types of TNBC

Six subtypes of TNBC were identified from the analysis of about 587 TNBC genes, including the high-expression growth factor pathway and myoepithelial markers basal-like 1 (BL1), basal-like 2 (BL2), mesenchymal-like (MES), mesenchymal/stem-like (MSL), immunomodulatory (IM), and luminal androgen receptor (LAR) (Yin et al., 2020). Recently, one study reported the molecular typing of TNBC which is the most popular and commonly referred as “Fudan typing” (Liu et al., 2016; Jiang et al., 2019). Using multi-omics sequencing, a research group categorized 465 TNBC specimens into four groups. Specifically, the MES type has enriched growth factor signaling pathways, the LAR type signals through androgen receptors, the IM type overexpresses immune cell and cytokine signaling genes, and the BL type uses reduced immune response genes to activate DNA repair and the cell cycle (Jiang et al., 2019).

### 2.1 Major classes of TNBC

#### 2.1.1 Basal-like (BL) subtype

BL tumors have high expression of proliferative genes and express ATR/BRCA pathways, which are involved in DNA damage response and accelerate cell proliferation (Ensenyat-Mendez et al., 2021). Since there is a lack of a repair system, polyADP-ribose polymerase 1 (PARP) inhibitors and platinum coordination complexes are widely accepted as treatment regimens for basal-like subtypes (Barchiesi et al., 2021). One study reported significant improvement in pathologic complete response (pCR) (58.7%) when carboplatin was used as an adjuvant compared to the non-carboplatin group (Von et al., 2014).

#### 2.1.2 Mesenchymal subtype

Mesenchymal tumors have higher levels of genomic instability, mutation burden, and copy number alterations than other subtypes. They are also associated with a lower number of immune cells and PDL1 expression, which indicate that these tumors have developed immune-evading mechanisms and contribute to their resistance to immunotherapy (Akhand et al., 2021).

Mesenchymal tumors are characterized by the alteration in growth factor signaling pathways and epithelial-to-mesenchymal transition (EMT). Due to phosphoinositide-3-kinase, catalytic, alpha polypeptide (*PIK3CA*) mutations or deficiency of phosphatase and tensin-homolog (PTEN), the mesenchymal subtype expresses the phosphoinositide 3-kinase/protein kinase B (PI3K/Akt) pathway and is sensitive to mammalian target of rapamycin (mTOR) inhibitors like NVP-BEZ235 (Zhao et al., 2020). EMT pathways can also be targeted by inhibiting the fibroblast growth factor receptor (Asleh et al., 2022).

#### 2.1.3 Luminal androgen receptor (LAR) subtype

LAR subtypes carry higher rates of ERBB2 mutations than other TNBC subtypes, despite being clinically classified as negative for HER2 genes (Lehmann et al., 2021). Estrogen/androgen metabolism pathways are differentially expressed in the luminal AR subtype. Therefore, inhibition of the luminal androgen receptor is a widely accepted treatment approach for this tumor subtype. Since *PIK3CA* mutations have also been reported in this tumor subtype, PI3K inhibitors can also be an effective therapeutic alternative (Ahn et al., 2016; Zagami and Carey, 2022).

#### 2.1.4 Immunomodulatory (IM) subtype

The IM subtype is characterized by the abundant expression of genes associated with immune cell processes. Genes and pathways of signal transduction linked to immune cells, include the NK cell, B cell receptor signaling, dendritic cell, T cell receptor signaling, interleukin (IL)-12/IL-7, and Th1/Th2 pathway, are substantially activated in the IM subtype. Patients with breast cancer of the IM subtype are advised to be treated with PD1, PDL1, CTLA-4, along with other immune checkpoint inhibiting agents (Yin et al., 2020). The medullary cancer of the breast and the IM subtype are nearly identical (Yin et al., 2020). The IM subtype’s immune signaling genes are significantly similar to the gene profile of medullary breast cancer, a unusual but histologically different kind of TNBC with high-grade histology linked to positive prognosis (Hubalek et al., 2017).

## 3 Epigenetic alteration in TNBC

### 3.1 ECM/EMT interplay–DNA methylation

The addition of a methyl group at the 5^th^ position of the pyrimidine ring to form 5-methylcytosine (5-mC) is known as DNA methylation, which generally occurs at genomic CpG sites (Kaleem et al., 2021b). DNA methylation is a heritable epigenetic phenomenon in normal mammalian growth that regulates gene activity (Maenohara et al., 2017; Zeng and Chen, 2019). Nevertheless, the abnormal methylation of CpG islands is a characteristic of numerous malignancies in the promoter domains of genes, which are involved in the upregulation of oncogenes and downregulation of tumor suppressor genes (Kagara et al., 2012). One of the major causes of disruptions in the cell cycle is epigenetics-associated DNA methylation. Under normal conditions, CpG islands that are short DNA sequences enriched with cytosine and guanosine remain in a non-methylated state. A family of enzymes known as DNA methyltransferases (DNMTs) facilitates the transfer of the methyl moiety from donor S-adenosylmethionine (SAM) to DNA. Mammalian DNMTs are mainly involved in the maintenance and *de novo* methylation. During embryogenesis, DNMT3A, and DNMT3B play a key role in the maintenance of *de novo* methylation, while, during DNA replication, maintaining the methylation patterns from parent to offspring (Mensah et al., 2021). DNA methyltransferases (DNMT1, 3A, and 3B) mediates DNA methylation modifications, leading to gene expression alterations. DNA methylation pattern overlaps between TNBC and other breast cancers due to similarity in the involved genes (Kaleem et al., 2021b; Uddin and Fandy, 2021; Zolota et al., 2021). The CpG islands undergo hypermethylation in TNBC (Stirzaker et al., 2015; Quintas-Granados et al., 2021). It has been reported that RB, CD44, p73, MGMT, and CDKN2B genes remain in methylated state, while MLH1, MSH6, MSH2, MSH3, DLC1, CACNA1A, GSTP1, CACNA1G, ID4, Twist1, and PMS2 genes remain in non-methylated state in TNBC. The two-pore domain potassium channels are differentially expressed in TNBC and the over-expression of KCNK5 and KCNK9 genes takes place due to hypomethylation of CpG island loci (Dookeran et al., 2017). In the miR-200b promoter region, MYC incorporates DNMT3A, which results in hypermethylation of CpG islands, followed by suppression of miR-200b, promoting mammosphere formation and EMT in TNBC cells (Pang et al., 2018). Some studies suggested that DNA methylation suppresses CREB3L1 and BRMS1 genes, which suppress metastasis (Ward et al., 2016; Xia et al., 2017).

Despite widespread hypomethylation, hypermethylation of specific genes has also been linked to TNBC (Sandhu et al., 2012). In TNBC, the methylation of several genes associated with the DNA damage response, including BRCA1 and 14-3-3σ (also referred to as HME1) has been reported (Yu et al., 2019). Reduced expression of BRCA1 mRNA is linked to methylation of the BRCA1 promoter. Investigations using cancer cell lines and tissues as models have shown that the decrease in gene expression is mediated by CpG island methylation of the 14-3-3σ gene. Moreover, methylation has also been observed in TNBC for cell-to-cell adhesion molecules, including E-cadherin, the inactivation of which could facilitate metastasis (Kashiwagi et al., 2010; Yu et al., 2019).

### 3.2 Histone modifications

After translation, the histone proteins undergo modifications, such as acetylation, ubiquitination, methylation, phosphorylation, and sumoylation at the N-terminal tails, which alters the structure of chromatin and gene transcription (Ramazi et al., 2020). Various levels of histone acetylation and methylation mark subclasses of breast tumors. The basal-like and HER2-positive tumors are the consequences of lysine acetylation (H3K18ac, H3K9ac, and H4K12ac) and methylation of arginine (H4R3me2) and lysine (H4K20me3, and H3K4me2) (Ramazi et al., 2020). In addition, the increased expression of H3K4ac and H3K4me3 has been suggested to modulate the EMT signaling pathway (Lin and Wu, 2020). The Brahma (BRM) expression levels are noted to be decreased in higher grade human breast cancer tissues than those of lower grade, as well as in triple-negative breast cancer cells (MDA-231), results in the loss of claudin group (Yang et al., 2019). A significant decline in the mRNA levels of eight HMTs, including PRDM6, SMYD3, EZH1, SETD7, SETD3, EHMT1, SETD1B, and PRDM4 and expression of PRDM15, SMYD2, SUV39H1, EZH2, SETD6, EHMT2/G9a, PRDM13, SMYD5, WHSC1, SUV39H2, SETDB, and SETD8 HMTs in basal-like tumor has been reported (Liu et al., 2015).

One study demonstrated a rise in H3K4ac and H3K4me3 markers in the metastatic MDA-MB-231 cell line compared to the mature MCF7 luminal cell line, emphasizing the variations amongst breast cancer subcategories (Messier et al., 2016). H3K4ac was believed to be involved in ER signaling in MCF-7 cells, while in the TNBC cell line (MDA-MB-231), H3K4ac and H3K4me3 are the EMT-linked genes that facilitate EMT-mediated signaling pathways (Zolota et al., 2021).

Histone deacetylases (HDACs) are generally used for various epigenetic treatments and regulate the tumor suppressor genes by removing acetyl groups from histones. Several solid and hematological malignancies have shown positive results, utilizing HDAC inhibitors (HDACi), inhibiting tumor growth and triggering apoptosis through various mechanisms (Mottamal et al., 2015). One study demonstrated that suberoyl anilide hydroxamic acid (SAHA, vorinostat) and sodium butyrate (NaB), both HDACIs, exhibit significant reductions in TNBC cell growth, induce cell cycle arrest at the G_0_/G_1_ phase, and trigger apoptosis. Furthermore, the phosphorylation, protein and mRNA levels of mutant p53 (mtp53) are reduced in TNBC cells by SAHA and NaB treatment (Wang et al., 2016). Panabinostat, another HDACi, has shown to induce hyperacetylation of histones H3 and H4 in TNBC cell lines, including MDA-MB-157, MDA-MB-231, MDA-MB-468, and BT-549, while reducing survival, proliferation, and apoptosis induction. Panabinostat has also been observed to reduce the growth of tumors in xenograft (MDA-MB-231 and BT549 cells) mouse models (Tate et al., 2012). Although knockdown of PTEN cells is resistant to these combinations, vorinostat has been noted to improve the growth inhibitory capacity of PARP inhibitor olaparib in TNBC cells (Min et al., 2015; Temian et al., 2018).

### 3.3 MicroRNA expression

MicroRNA (miRNAs), 20–22 nucleotides long, are small single-stranded non-coding RNAs that control gene expression through post-translational silencing of the target genes. miRNAs have been suggested as crucial regulators of carcinogenesis and target different aspects of the TNBC microenvironment with increased efficacy (Qattan, 2020). MicroRNA expression can be monitored through various mechanisms, such as single nucleotide polymorphisms (SNPs), chromosomal abnormalities, and mutations in the primary transcripts (Lu et al., 2005). The significant decrease in the miR-200b/a/c expression through blocking PKC and targeting BMI1 proteins, (SIP1 and ZEB1/2), leads to down-regulation of EMT and suppresses TNBC cell proliferation (Piasecka et al., 2018). One study reported that the up-regulation of miR-200b suppresses the migration of MDA-MB-231 TNBC cells (Piperigkou et al., 2020). In addition, miR-200 and miR-205 suppress EMT and cancer metastasis through the hypermethylation of the ZEB2 and ZEB1 promoter regions (E-cadherin transcriptional repressors). By targeting the RabGTPase family 27a and MMP 11, MiR-145 suppresses cell proliferation, decreases cell-cell adhesion, and facilitates apoptosis (Tang et al., 2016; Piperigkou and Karamanos, 2019). Moreover, the overexpression of miR-155 in TNBC provides a protective effect and enhances patient survival through DNA-damaging pathways. Phosphatase and Tensin homolog (PTEN) and pro-apoptotic phosphatase could be miR-21 targets (Gasparini et al., 2014). Among miRs that are markedly elevated in TNBC is miR-27a/b and scientific studies have linked its high expression to a poor overall patient survival rate. On the other hand, the low expression of miR-30a has been linked to higher lymph node metastases and histological grade in TNBC patients. In addition, miR-30a has also been noted to inhibit TNBC cells’ epithelial-mesenchymal transition (Wang X. et al., 2018). A poor prognosis of TNBC has been linked to miR-210 elevated levels and low expression of miR-210 increases disease-free survival and overall survival (Wu, 2020; Santana et al., 2022). TNBC cell invasion and migration are facilitated by miR-454, which subsequently promotes TNBC cell proliferation. By controlling the levels of caspase 3/7 and the anti-apoptotic protein BCL-2, miR-454 suppresses radiation-induced apoptosis in TNBC cells. The elevated levels of miR-454 have also been shown to alter the expression of pAKT and PTEN in TNBC cells (Li et al., 2017). There is a poorer chance of survival for patients with elevated miR-146a levels compared to those having low expression levels. Moreover, a poorer prognosis is associated with elevated levels of miR-146a in TNBC, indicating that the miR-146 expression is an independent variable affecting patient prognosis (Santana et al., 2022).

### 3.4 Long non-coding RNAs

**FIGURE 1:**
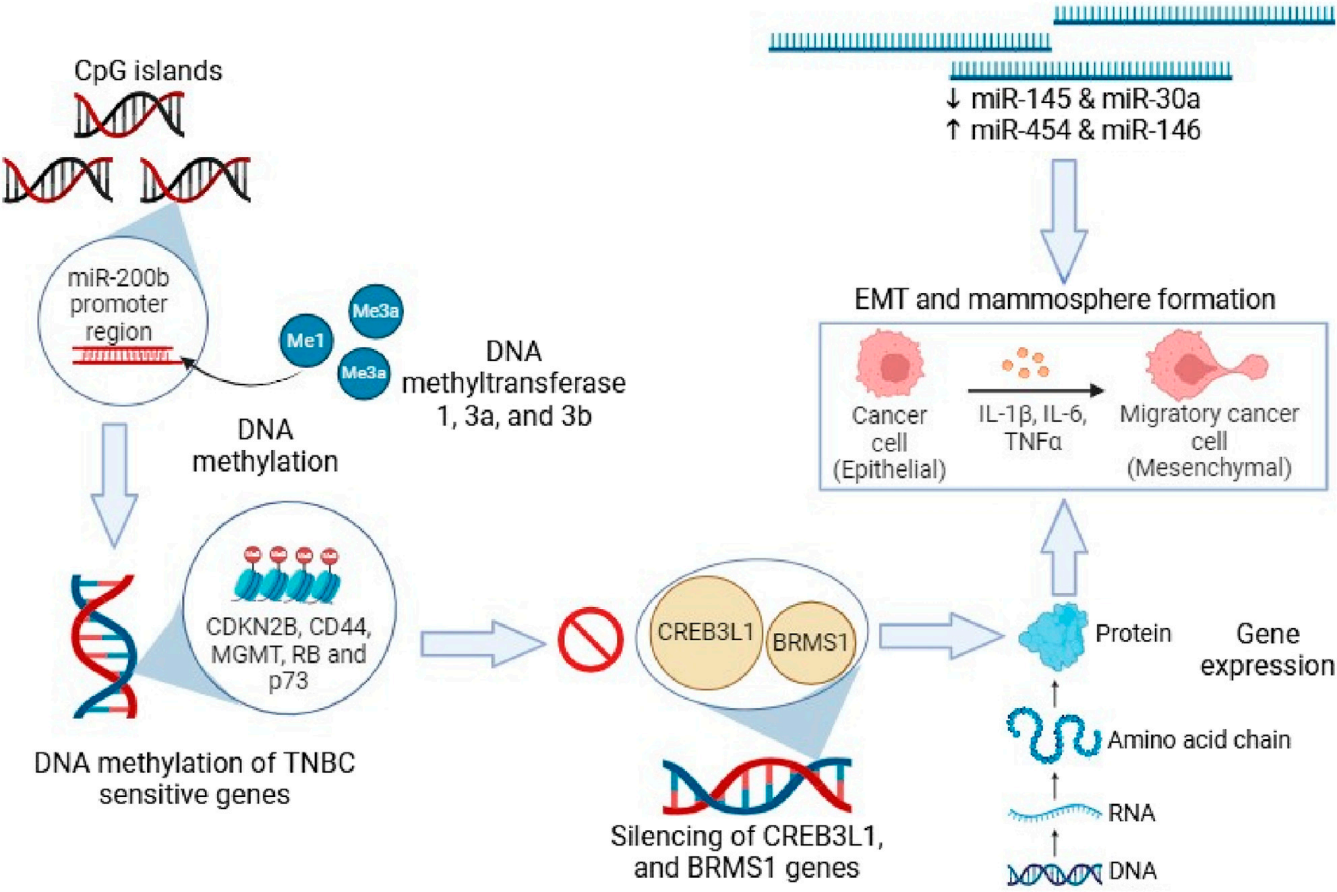
DNA methylation on TNBC susceptible genes and cellular migration.

Long non-coding RNAs (lncRNAs) are non-protein-coding transcripts and <200 nucleotides. Several studies reported dysregulated lncRNA expression through a variety of mechanisms in numerous kinds of tumors, such as ovarian, breast, hepatocellular carcinoma, and others (Tripathi et al., 2018; Wong et al., 2018). Furthermore, several lncRNAs have also been reported to play a vital role in different biological processes, such as invasion, differentiation, apoptosis, proliferation, and cell development. LncRNAs have been suggested to interfere with miRNA-mediated gene regulation by acting as miRNA “sponges” and competing with miRNA-targeted mRNAs. Molecular studies have demonstrated that the primary molecular mechanism of lncRNA biological function is competing endogenous RNA processes and network formation, which sequesters miRNAs while preventing post-translational regulation of protein-coding counterparts. Some lncRNAs associate with mRNAs to enhance and protect against the damaging effects of miRNA (Zhang W. et al., 2021). Growth arrest and DNA-damage-inducible alpha is regulated by lncRNA TARID (TCF21 antisense RNA-induced demethylation), which in turn stimulates tumor suppressor transcription factor 21 (TCF21) through promoter demethylation (Arab et al., 2014; Aram et al., 2017). HOX transcript antisense RNA, a lncRNA binds to polycomb repressive complex 2, causes the trimethylation of H3 lysine 27 (H3K27me3) of the homeobox D cluster locus, thereby promoting epigenetic silencing (Fang and Fullwood, 2016). To protect from antitumor cell metastasis, the natural antisense transcript (NAT) of zinc finger E-box binding homeobox 2 ZEB2 binds to an intronic 5′ splice site that prevents ZEB2 mRNA from interweaving (Huarte, 2015). Some lncRNAs function as tumor suppressors, whereas others function as oncogenes (Qi and Du, 2013). In hepatocellular carcinoma, the metastasis and epithelial-to-mesenchymal transition (EMT) are mediated by TGF-β activated lncRNA (lncRNA-ATB). NF-κB-with lncRNA interconnection functions as a scaffold to inhibit phosphorylation of IκB, which activates the tumorigenic NF-κB pathway (Peng et al., 2017). The suppressor of KAI1 in breast cancer inhibits the KAI1/CD82 metastasis suppressor gene and is known as metastasis-inducing lncRNA, which is upregulated in TNBC (Arab et al., 2014; Aram et al., 2017). *In vitro* and *in vivo* studies revealed suppression of tumor proliferation in the LINC01638 knockdown model since LINC01638 facilitates EMT via the Twist1 expression (Luo et al., 2018). Moreover, LncRNA, such as LUCAT1, PAPAS, and HCP, have shown to facilitate TNBC by altering miR-34a, miR-219a-5p, and miR-5702 miRNAs (Wang L. et al., 2019; Kong et al., 2019; Zhan et al., 2020). Apoptosis and cell proliferation suppression have been enhanced with lncRNA rhabdomyosarcoma 2-associated transcript (RMST) (Wang L. et al., 2018). The overexpression of LncRNA NEF inhibit migration and invasion of TNBC cells (Song et al., 2019). Similarly, LncRNA XIST block EMT via modulation of miR-454 in TNBC cells *in vitro* and *in vivo* models (Li et al., 2020; Zolota et al., 2021), as depicted in Figure 1. In the following section, we have covered different natural compounds that have shown encouraging results for the treatment of TNBC and highlight their potential through various mechanistic pathway targeting.

## 4 Natural compounds for the treatment of TNBC

### 4.1 Epigallocatechin-3-gallate (EGCG)

Epigallocatechin-3-gallate (EGCG), a green tea-derived polyphenol, exhibits anti-inflammatory, antioxidant, and antitumor activities (Alserihi et al., 2021; Mokra et al., 2022; Alserihi et al., 2023; Suhail et al., 2023). It inhibits the catechol-o-methyltransferase and DNA methyltransferase, thereby reducing the proliferation of the TNBC cells. It also causes the re-expression of the RAR-B, MGMT, and CDKN2A genes (Schröder et al., 2020). EGCG has been stated to upregulate the CASP1, CASP3, and CASP4 levels and decrease TP53 and FAS expression, repressing TNBC cell growth and proliferation (Bimonte et al., 2020). EGCG enhances the chemotherapeutic effect through β-catenin, EGFR, PI3K/Akt, MAPK, and NF-κβ pathways (Bimonte et al., 2020). Anti-apoptosis genes, such as induced myeloid leukemia cell differentiation protein (MCL1) and insulin-like growth factor-1 receptor (IGF1R) are reported to be downregulated and the upregulation of Receptor-interacting protein kinase 2 (RIPK2), Bcl2-associated athanogene 3 (BAG3), and X-linked inhibitor of apoptosis protein (XIAP) in response of EGCG treatment has also been noted (Ensenyat-Mendez et al., 2021). Expression of Golgi membrane protein 1 (GOLM1), metastasis promoter protein, has also been suppressed by EGCG in MDA-MB-231 cells through the HGF/HGFR/AKT/GSK-3/β-catenin/c-Myc signaling pathway (Xie et al., 2021).

### 4.2 Thymoquinone (TQ)

Thymoquinone (2-methyl-5-isopropyl-1,4-benzoquinone) is isolated from black cumin (*Nigella sativa*) seeds. It has various pharmacological properties such as antioxidant, anti-inflammatory, anti-diabetic, and immunomodulatory effects (Kabil et al., 2018). Several investigations have shown that TQ exhibits therapeutic potential against different categories of cancer via a variety of mechanisms, such as the production of reactive oxygen species, induction of apoptosis, cell cycle arrest, suppression of angiogenesis, and cell proliferation (Banerjee et al., 2010; Abdullah et al., 2021; Kaleem et al., 2024). It can modulate numerous signaling targets/pathways involved in cancer, such as STAT3, PPAR-γ, NF-κB, and p53. TQ reduces the level of the eEF-2K protein and important downstream targets such as Src/FAK and Akt which are therapeutically significant. Additionally, TQ activates the tumor suppressor miR-603 in association with suppression of NF-kB, resulting in a notable reduction in TNBC cell migration, invasion, proliferation, and colony formation (Gomathinayagam et al., 2020). Administration of TQ (20 and 100 mg/kg) has shown a marked reduction in the growth of MDA-MB-231 tumors and prevented eEF-2K activity (Kabil et al., 2018). The anticancer potential of TQ is manifested through DNA methylation (DNMT1 dependent) in leukemia cells (Pang et al., 2017). The treatment of TQ results in the down-regulation of really interesting new gene (RING) finger domains 1 (UHRF1), HDAC1/4/9, KDM1B, G9A, DNMT1/3A/3B, and KMT2A/B/C/D/E, and ubiquitin-like containing plant homeodomain (PHD) (Ashraf et al., 2017; Qadi et al., 2019; Abdullah et al., 2021). One study observed that administration of TQ decreases the mRNA expression of TWIST1 (EMT-promoting transcription factor) in MDA-MB-435 cell lines (Khan et al., 2015).

### 4.3 Curcumin

Curcumin (1,7-bis (4-hydroxy-3-methoxyphenyl)-1,6-heptadiene-3,5-dione), also known as diferuloylmethane is a polyphenolic compound present in the rhizomes of *Curcuma longa*. In Asian nations, curcumin has been conventionally used as a medical herb because of their anti-inflammatory, antimutagenic, antimicrobial, antioxidant, and anticancer properties (Hewlings and Kalman, 2017; Ahmad et al., 2024). Scientific studies have reported the anticancer effects of curcumin against TNBC cells via EGFR tyrosine kinase, PI3K-Akt, Foxo, JAK-STAT, and HIF-1 signaling pathways. It also modulates PTGS2, EP300, STAT3, TNF-α, GSK3B, PPARG, AKT1, EGFR, NFE2L2, and MMP-9 genes expression and shows anti-proliferative activity. It has been reported to prevent the invasion and proliferation of the MDA-MB-231 cells and induce apoptosis (Deng et al., 2022). A study revealed that curcumin blocks the invasion, proliferation, migration, and mammosphere formation in TNBC cells (Li M. et al., 2022).

Curcumin has also been reported to successfully inhibit TNBC growth by preventing the protein salt-induced kinase-3 (SIK3) expression in tumor-derived xenograft mice from patients (TNBC-PDX). Tumor tissues exhibit a notably elevated SIK3 mRNA expression (73.25 times, n = 183) compared to the neighboring normal tissues. Curcumin (>25 μM) could prevent SIK3-mediated cyclin D overexpression, which arrests the G1/S cell cycle and suppresses the growth of TNBC (MDA-MB-231) cancer cells. During EMT, there is a correlation between the overexpression of SIK3 and higher levels of mesenchymal markers, such as, Twist, MMP3, α-SMA, and Vimentin. Curcumin substantially attenuates tumor migration by inhibiting SIK3-mediated EMT (Cheng et al., 2021).

### 4.4 Calycosin (CA)

Calycosin (CA) is a flavonoid (7,3ʹ-dihydroxy-4ʹ-methoxyisoflavone, C16H12O5) phytoestrogen obtained from *Astragalus membranaceus* and has shown anticancer activity especially against TNBC cells. It is also found in plants, such as *Hedysarum Polybotrys Hand.-Mazz., Puerarialobata* (Wild.) *Ohwi, Pterocarpuserinaceus Poir., Caraganaacanthophylla Kom.*, *Sophoraflavescens Aiton*, *Glycyrrhizauralensis Fisch*, and *Millettialaurentii* De Wild (Deng et al., 2021). In the ER- and PR-negative subtype, higher expression of ER-α30 has been reported in BT-549 and MDA-MB-231 TNBC cell lines. ER-α30 expression has been noted to be blocked by CA along with the inhibition of TNBC metastasis (Li et al., 2023). Calycosin reduces the migration, invasion, and proliferation of breast tumor cell lines (T47D and MCF-7) in a time- and dose-dependent manner. In addition, basic leucine zipper ATF-like transcription factor (BATF) elevate TGFβ1 mRNA and protein levels, stimulating breast cancer cell migration and invasiveness. Treatment with CA inhibits EMT of breast cancer cells via decreasing levels of TGFβ1 and BATF while up-regulating the expression of E-cadherin and decreasing the expression of vimentin, CD147, MMP-2/9, and N-cadherin. Furthermore, CA increases invasiveness and migration when incubated with TGFβ1, while knockdown of TGFβ1 decreases these properties in BATF-overexpressing breast cancer cells. These findings indicate that CA prevent EMT and the growth of breast cancer cells via reducing BATF/TGFβ1 signaling (Zhang Z. et al., 2021). In conclusion, CA prevents the growth and metastasis of TNBC by targeting the ER-α splice variant ER-α30 and reducing its expression in cancer tissues (Li et al., 2023).

### 4.5 Mango (*Mangifera indica*)

Mangois obtained from *Mangifera indica L.*, family Anacardiaceae, a key South and Southeast Asia (Kumar et al., 2021). The mango leaf extracts have various biological properties, including lipid-lowering, liver protection, anti-diarrheal, antioxidant, anti-microbial, anti-cancer, and anti-diabetic effects (Kumar et al., 2021). Extracts from the leaves, seeds, and bark of *M. indica* have been used as pyruvate kinase M2 (PKM2) inhibitors. The extract of *M. indica* bark and seeds have shown growth inhibition through PKM2 blockage in the MDA-MB231 cells, indicating its potential for the management of TNBC (Rasul et al., 2021).

### 4.6 Cordycepin

Cordycepin (CD) is a 3′-deoxyadenosine analog isolated from the caterpillar fungus *Cordyceps militaris* (Radhi et al., 2021). It is one of the major constituents found in Cordyceps mushrooms. The CD has various pharmacological properties, including antiviral, antitumor, immunity regulation, lipid-lowering, antiplatelet agglutination, antibacterial, and memory improvement (Liu X. C. et al., 2019). CD can inhibit the expression of SLUG and TWIST, thereby inactivating the EMT signaling pathway. In addition, cordycepin significantly downregulates the expressions of *ZEB2*, *TWIST1*, *ZEB1*, *SLUG*, and *SNAIL*, which are responsible for the inhibition of EMT in 4T1 and BT549 cells (Wei et al., 2022). The Hedgehog pathway has been suggested as the most prominent pathway in breast cancer. One study reported that cordycepin inhibits the Hedgehog pathway and suppresses growth and metastasis in the MDA-MB-231-induced mice xenograft model. The CD has been reported to markedly reduce the expression levels of the various components linked to apoptosis, metastasis, and growth in TNBC. Therefore, cordycepin could be a novel lead compound for TNBC therapy via Hedgehog pathway regulation (Wu et al., 2022).

### 4.7 Ailanthone (AIL)

Ailanthone (AIL) is a quassinoid extract isolated from *Ailanthus altissima* and a pentacyclic diterpene lactone compound belongs to Simaroubaceae family (Ding et al., 2020). It has shown antimalarial, anti-HIV, anti-microbial, anti-ulcer, anti-inflammatory, anti-allergic, anti-tuberculosis and antitumor properties (Wang R. et al., 2018; Ding et al., 2020). The bone marrow-derived macrophages (BMMs) are usually activated by the receptor activator of nuclear factor-κB nuclear ligand-receptor activator (RANKL), and MDA-MB-231 cell conditioned medium. These findings revealed a substantial reduction in the expression of osteoclast-forming genes and inhibition in infiltration and migration of MDA-MD-231 cells at low concentrations. AIL upregulates Forkhead box protein 3 (FOXP3) expressions while suppressing IL-1β and RANKL expression. AIL has also been reported to inhibit NF-κB, PI3K/protein kinase B (Akt), and MAPK signaling pathways (Wang et al., 2023).

### 4.8 Polyphyllin III/dioscin (DS)

Polyphyllin III is the main saponin obtained from *Paris polyphylla* rhizomes. The combination of polyphyllin III and sulphasalazine sensitizes TNBC cells by enhancing ferroptosis and lipid peroxidation. Ferroptosis through ACSL4-mediated lipid peroxidation is the primary mechanism of polyphyllin III anticancer activity (Zhou et al., 2021). One study reported a considerable decrease in invasion, colony formation ability, and cellular migration in MDA-MB-231 and MCF-7 cells in response to DS treatment. DS dramatically reduce the development of mammospheres in a dose-dependent manner during the process of differentiation triggering the manifestation of breast cancer stem-like cells. It also elevates p21 and p53 levels in breast cancer stem-like cells while decreasing cyclin B1 and cdc2 levels in MDA-MB-231 cells and cyclin D/E and CDK2/4 levels in MCF-7 cells. The administration of DS has also been noted to arrest cell cycle at G0/G1 and G2/M in MDA-MB-231 and MCF-7 cells, respectively. Additionally, DS has been noted to downregulate p-mTOR and p-Akt expression and increases p38 phosphorylation. These findings indicate that DS regulates the Akt/mTOR and p38 mitogen-activated protein kinase signaling pathways to inhibit the growth of breast cancer stem-like cells by inducing cell cycle arrest (Ock and Kim, 2021).

### 4.9 Tanshinone IIA

The lipophilic (Tanshinone I, IIA, IIB; cryptoTanshinone; other associated compounds) and hydrophilic (polyphenolic acids, danshensu, protocatechuic aldehyde, and protocatechuic acid) are the two most common active compounds obtained from the rhizomes and roots of *Salvia miltiorrhiza* (Labiatae family). Tanshinone IIA (Tan IIA), a diterpene quinone has been reported to have various therapeutic benefits against different diseases (Shang et al., 2012; Fang et al., 2021). It can target VEGF and mTOR/p70S6K/4E-BP1 signaling pathway and can inhibit VEGF and HIF-1α expression, which results in angiogenesis suppression (Li et al., 2015). Tan IIA prevents the total protein kinase C (PKC) activity, particularly PKC isoforms ζ and ε. In addition, Tan IIA prevents the Ras/MAPK pathway, which is tightly regulated by PKC signaling. Furthermore, Tan IIA trigger autophagy and cell cycle arrest and dramatically affect PI3K/Akt/mTOR signaling (Lv et al., 2018).

### 4.10 Glyceollins

Soybean contain a high amount of isoflavonoids and belongs to the Papilionoideae subfamily. It is one of the major sources of oil and protein globally (Yue et al., 2023). Glyceollins are a group of phytoalexins, the main constituents in soybean and a secondary isoflavones metabolite (Lecomte et al., 2017). It is produced when exposed to fungi and in the presence of some abiotic elicitors, such as aluminum chloride, methyl jasmonate, and UV light (Park et al., 2017). Glyceollin I, II, and III are synthesized by the *de novo* pathway from the soy isoflavone daidzein. It is also reported to suppress the proinflammatory cytokines by inhibiting NF-κβ phosphorylation (Pham et al., 2019). Inhibition of the P13K/Akt/mTOR pathway and blocking of HIF-1 synthesis has been reported as one of the mechanism of its action for suppressing tumor growth in TNBC (Rhodes et al., 2012; Lee et al., 2015). Based on these studies, glyceollins seems to be promising therapeutic against triple-negative breast cancer.

### 4.11 Amentoflavone

Amentoflavone is a phenolic biflavonoid extracted from the leaves of *Selaginella tamariscina* (Imran et al., 2019). Anticancer activity of amentoflavone is brought through mediating nuclear factor kappa-B (NF-κβ), extracellular signal-regulated kinase (ERK), and PI3K/Akt pathway (Xiong et al., 2021). Amentoflavone has shown to block the Hedgehog signaling pathway by decreasing the expression of Gli1, OCT4, NANOG, and Smo. It also inhibits tumor sphere formation and controls the expression of different stem cell markers (CD24, ALDH1, and CD44) in a dose-dependent manner. The knockdown of Gli1 via siRNA leads to decreased formation of tumor sphere effectiveness in SUM159 breast cancer stem cells and dramatically affects ALDH1, OCT4, NANOG, and CD24/44 expression. These results suggest that amentoflavone suppresses the stemness of SUM159 breast cancer via targeting Hedgehog/Gli1 signaling, highlighting it as a promising molecule for the management of breast cancer stem cells (Bao et al., 2019).

### 4.12 Sulforaphane (SFN)

Sulforaphane [1-isothiocyanato-4-(methylsulfinyl) butane] belongs to the isothiocyanate class of phytochemicals. Glucoraphanin, a glucosinolate precursor of SFN, is a glucosinolate found in cruciferous vegetables, such as broccoli, cabbage, cauliflower, and kale (Kim and Park, 2016). The MDA-MB-468 cells treated with SFN showed a reduction in mTOR and inhibition of Akt activity. SFN significantly suppresses the PI3K/Akt/mTOR pathway within cells where the downstream pathway is overactivated due to the loss of PTEN and EGFR upregulation. EGFR upregulation and PTEN reduction are common in TNBC and are linked to aggressive behavior and a poor prognosis of TNBC patients (Beg et al., 2015). SFN exerts growth-inhibitory activity against the MDA-MB-468 TNBC cell line, which shows the activation of the PI3K/Akt/mTOR signaling pathway and substantial rise in EGFR expression along with simultaneously suppression of phosphatase and tensin homolog (PTEN). These investigations indicated that SFN may serve as novel targeted therapeutic for treating TNBC, which is characterized by an overactive signaling pathway downstream of EGFR (Yasunaga et al., 2022).

### 4.13 Fisetin

Fisetin (3,3′,4′,7-tetrahydroxyflavone) is a bioactive flavonol molecule found in fruits and vegetables, such as strawberries, apples, persimmon, grapes, onion, cucumbers and has shown anticancer activity (Imran et al., 2020; Kubina et al., 2021). It is a potent blocker of matrix metalloproteinase (MMP)-1 activity, an enzyme essential for the remodeling and deterioration of the extracellular matrix that plays a significant role in cancer development. By inhibiting urokinase plasminogen activator, fisetin decrease angiogenesis, and inhibit tumor growth (Khan et al., 2013). It activates the caspase-3/8-dependent pathway through ERK1/2, Ca^2+^-dependent endonuclease, and caspase-3. Fisetin has also been reported to block mitogen-activated protein kinases (MAPK) and PI3K/Akt/mTOR signaling pathway (Chamcheu et al., 2019). These studies indicate the possible agonistic activity of fisetin on TNBC metastasis, which reversed EMT by blocking the PTEN-Akt-GSK-3β signaling pathway. These findings demonstrated that fisetin could be a novel, potentially effective therapy for metastatic breast cancer in TNBC patients (Li et al., 2018).

### 4.14 Ginsenoside 20(S)-protopanaxadiol

Ginsenoside is the active ingredient of American/Asin ginseng, isolated from rhizomes and roots of *Panax ginseng/notoginseng* of Araliaceae family. It has been reported to inhibit invasion and metastasis. The main constituents present in Panax herbs that exert an anticancer effect include PTS (20(S)-protopanaxatriol (PPT) saponins) and PDS (20(S)-protopanaxadiol (PPD) saponins) (Chen et al., 2016). PPD, one of the major active metabolites from *P. ginseng*, has been widely reported to possess pleiotropic anticancer activities against various cancers (Peng et al., 2019). A low concentration of PPD (<20 μM) has been reported to reduce the metastatic potential of SUM159 and MDA-MB-231 cells by directly inhibiting cell motility, invasiveness, and adhesion. In contrast, PPD at a dose of >30 μM has been observed to show decreased cell proliferation and induced apoptosis through cell cycle arrest at the G_0_/G_1_ phase. In addition, PPD therapy has also been observed to significantly reduce tumor growth in TNBC xenograft models. PPD induced the expression of tissue inhibitors of metalloproteinases (TIMPs) and reverts the EMT while reducing the expression and functioning of matrix metalloproteinases (MMPs). Through agonists or inhibitors of the EGFR and MAP kinases family, PPD therapy further confirmed the reduction in EGFR phosphorylation and down-regulation of ERK1/2, p38, and JNK signaling activation. These studies highlight PPD as a novel lead compound for the therapy of TNBC cells via the downregulation of the EGFR-mediated MAPK signaling pathway (Peng et al., 2019).

### 4.15 Quercetin

Quercetin is a flavonol mainly extracted from onion, which is present in the form of Que glycoside and has antioxidant properties (Salehi et al., 2020). Cherries, grapes, mangoes, citrus fruits, apples, plums, and buckwheat contain some quercetin (Kim et al., 2019; Wang et al., 2022). Quercetin can target insulin-like growth factor-1 receptor (IGF1R), Erk1/2 and inhibits the expression of EMT transcription factors Snail and Slug, thereby blocking the TNBC cell metastasis. It can activate IGF1R and its downstream kinases, such as Akt and Erk1/2, to prevent growth in human MDA-MB-231 breast cancer cells in a dose-dependent manner. It also inhibits the autocrine or paracrine loop of IGF1 signaling by increasing the production of IGF-binding protein-3 and decreasing the secretion of IGF1 in the culture medium of MDA-MB-231 cells. Quercetin has also been noted to inhibit the growth of MDA-MB-231 tumor xenografts mouse models through deactivation of IGF1R, upregulation of epithelial markers (keratins 18 and 19), and decreased levels of mesenchymal markers (Snail, Slug, fibronectin, and vimentin) (Chen et al., 2021).

### 4.16 Luteolin

Luteolin, (3′,4′,5,7-tetrahydroxyflavone), belongs to a group of naturally occurring compounds called flavonoids. It is present in high concentration in chrysanthemum flowers, onions, parsley, carrots, celery, broccoli, and bell peppers and have shown several biological activities, such as neuroprotective, antidiabetic, anticancer, antiallergic, anti-inflammatory, and anti-hypertensive properties (Çetinkaya and Baran, 2023). By inducing the expression of E-cadherin, causing shrinkage of the cytoskeleton, and downregulation of N-cadherin, vimentin, and snail, luteolin can reverse EMT. Its anticancer activity is through the modulation in the expression of ER stress-associated proteins, activation of reactive oxygen species (ROS), and mitochondrial dysfunction (Imran et al., 2019). Luteolin can block VEGF, which suppresses the β-catenin pathway, thus inhibiting proliferation and mammosphere formation in TNBC. Low luteolin dose significantly inhibits VEGF secretion in MDA-MB-231 (4175) LM2 cells (Cook, 2018). *In vitro* and *in vivo* experiments reported that luteolin induces suppression of SGK1, increases the translocation of FOXO3a into the nucleus, and subsequently transcribes BNIP3, which in turn enables BNIP3 to interact with autophagy and apoptosis proteins. These studies highlight that luteolin promotes apoptosis and autophagy in TNBC using the SGK1-FOXO3a-BNIP3 pathway, suggesting its potential against TNBC therapy (Wu et al., 2023).

### 4.17 Astragalus polysaccharide (APS)

*Astragaliradix* is an important tonifying herb, derived from the dried root of *A. membranaceus* (Fisch.) Bge. (family Leguminosae) and used for anemia, weakness, allergic reactions, etc. (Guo et al., 2019). Research has shown many biological properties of APS, including anti-inflammatory, antioxidant, immunoregulatory, and antitumor (Zhao et al., 2018). APS significantly inhibits the expression of PIK3CG, Akt, and Bcl-2 and substantially suppresses the activity, invasion, and apoptosis in MDA-MB-231 cells. Therefore, APS could be a promising therapeutic compound for the management of TNBC (Liu C. et al., 2019).

### 4.18 4β-Hydroxywithanolide E (4-HW) and withaferin A (WA)

*Physalisperuviana* (golden berry) is an edible plant from the Solanaceae family. 4β-hydroxy withanolide E (4HW) is a natural compound isolated from an edible plant, *Physalisperuviana*. The 4bHWE withanolides are plant-derived C (28) steroidal lactones with potent anti-cancer properties (Chiu et al., 2013). Scientific studies have reported the anticancer potential of 4HW against prostate, oral, lung, and breast cancer (Tang et al., 2018; Hsieh et al., 2021). Similarly, withaferin A, [(4β,5β,6β,22*R*)-4,27-dihydroxy-5,6:22,26-diepoxyergosta-2,24-diene-1,26-dione] is a 28 carbon-containing withanolides with ergostane framework and a δ-lactone (Sultana et al., 2021). It is a steroidal lactone extracted from *Withaniasomnifera L.*, also known as Ashwagandha (Dutta et al., 2019; Paul et al., 2021). WA has been reported to inhibit NF-κβ signaling, Hsp90 chaperone, angiogenesis, cell growth, and trigger apoptosis. On the other hand, 4-HW prevent the growth of cancer cells via inducing ROS-mediated DNA damage (You et al., 2014) and by binding directly to HSP90 molecules, which is comparable to the effects of WA on cancer cells (Wang et al., 2012). The withanolides 4-HW and WA induce apoptosis/necrosis and results in cell cycle arrest, significantly reducing TNBC cells’ viability. The PI3K/Akt pathway has been noted to be modulated in response to 4-HW and WA. These findings reveal that withanolides may have potential in the management of TNBC (Wang H. C. et al., 2019).

### 4.19 Fucoidan

Fucoidan is isolated from marine sources, such as brown algae, sea cucumbers, *Sargassum stenophyllum*, and *Fucus vesiculosus* (Luthuli et al., 2019; Mansour et al., 2019). It exhibits potent biological activities, such as immunomodulatory, antivirus, antithrombotic, anticoagulant, and anti-tumor activity (Ale et al., 2011). It has been reported to downregulate the expression of vimentin and N-cadherin (mesenchymal markers) while upregulating E-cadherin and zonula occludens-1, which suppresses migration of MDA-MB-231 cells. Fucoidan treatment significantly reduce the HIF-1α protein expression through HIF1-α signaling pathway (Li et al., 2019). Therefore, fucoidan may be a promising therapeutic for treating TNBC through downregulating the HIF1-α signaling pathway.

### 4.20 Arctigenin

Arctigenin (Atn) is a bioactive lignan obtained from the seeds of *Arctium lappa L*. (Batool et al., 2022). Cancerous inhibitor of protein phosphatase 2A (CIP2A) is a molecular marker in human cancer that inhibits the tumor-suppressor protein, protein phosphatase 2A (PP2A), and subsequently increases cancerous growth (Alzahrani et al., 2020). Arctigenin activates the PP2A and downregulates CIP2A, which phosphorylates and inhibits the Akt pathway, suppressing TNBC cell metastasis (Huang et al., 2017). It prevent MDA-MB-231 and BT549 cells from proliferation, migrating, and invasion and from undergoing EMT through inhibition of 4EBP1 (Luo et al., 2021). On the other hand, the upregulation of 4EBP1 might counteract the inhibitory effects of Atn on proliferation, migration, invasiveness, and EMT.

### 4.21 Propolis and EGCG

Propolis is a natural resinous mixture produced by honeybees from the substances collected from plants, buds, and exudates (Wagh, 2013). It contains caffeic, dihydrocinnamic, and p-coumaric acids. Treatment with p-coumaric acid and EGCG significantly reduce the cell viability of four triple-negative breast cancer cell lines (BT-20, BT-549, MDA-MB-231, and MDA-MB-436) through DNA methyltransferase targeting. Propolis and EGCG have been reported to demethylate the promoter region of RASSF1A in BT-549 cells highlighting their potential against TNBC (Assumpção et al., 2020).

### 4.22 Sanguinarine (SANG)

Sanguinarine is a benzophenanthridine alkaloid, isoquinoline derivative, derived from rhizomes of the plant species *Sanguinaria canadensis*. This alkaloid is present as neutral alkanolamine and cationic iminium forms. Sanguinarine [13-methyl (1,3) benzodioxolo (5,6-c)-1,3-dioxolo (4,5) phenanthridinium] affects the Na^+^-K^+^-ATPase transmembrane protein, which leads to death of animal cells (Singh and Sharma, 2018). It is an excellent DNA and RNA intercalator where only the iminium ion binds (Basu and Kumar, 2016). SANG has been noted to inhibit Akt expression and upregulated the mRNA expression of Bcl-2, TNFRSF, and CASP in MDA-MB-468 cells, resulting in induced apoptosis caused by S-phase cell cycle arrest (Messeha et al., 2023). The mechanism underlying the antiangiogenic potential of SANG is TNF-α-mediated in, MDA-MB-231, and MDA-MB-468 cells. Furthermore, SANG block the NF-κB, ERK1/2 signaling pathway, and the CCL2 regulator gene IKBKE (Messeha et al., 2022).

### 4.23 Eupalinolide J (EJ)

Eupalinolide J is a sesquiterpene lactone, the main constituent in *Eupatorium lindleyanum* and popularly known as Yemazhui (Wu et al., 2020; Hu et al., 2023). It inhibits cancer cell growth by targeting the Akt and STAT3 signaling pathway (Lou et al., 2019). EJ suppresses the MMP-2/9 protein expression and inhibits metastasis. It inhibits TNBC cell proliferation by inducing cell apoptosis, hindering the mitochondrial membrane potential (MMP), and arresting the cell cycle.

### 4.24 Ursolic acid (UA)

Ursolic acid is a natural pentacyclic triterpene compound present in various fruits and vegetables. It is obtained from the leaves of several plants (rosemary, marjoram, lavender, thyme, and organum), fruits (apple fruit peel), flowers, and berries. UA has shown varied pharmacological activities, such as antioxidant anti-apoptotic, anti-carcinogenic, and anti-inflammatory properties. It inhibits cancer cells by inhibiting nuclear factor-kappa B signaling pathways (Seo et al., 2018). UA can dramatically suppress the growth, migration, invasion, and induction of cell cycle arrest and apoptosis in MDA-MB-231 and MDA-MB-468 cells in a concentration-dependent manner. The administration of UA has also been noted to result in a significant decrease in the tumor body and tumor weight of TNBC-xenograft mice, indicating its potential *in vivo*. One study observed that UA may inhibit the p53 signaling pathway, potentially reducing TNBC cell proliferation and decreasing the expression of PLK1 and CCNB1 proteins (Zhang Y. et al., 2021). These results provide compelling evidence that UA intervention with multi-target treatment effectively treats TNBC. Hence, UA could be a promising therapeutic for the treatment of TNBC.

### 4.25 Morin

The flavonoid morin, isolated from the Moraceace family, has shown numerous pharmacological properties, such as antioxidant, anti-inflammatory, and anti-cancer activity (Solairaja et al., 2021; Balaga et al., 2023). Morin has been reported to alter Kelch-like ECH-associated protein1/Nuclear erythroid-2-related factor (Keap1/Nrf2), MAPK pathways (Tian et al., 2017), and Wnt/β-catenin and induces apoptosis (Balaga et al., 2023). Inhibition of FOXM1 and attenuation of EGFR/STAT3 signaling pathways has been observed when a combination of morin and doxorubicin (Dox) is used. Phosphorylation of STAT3 and EGFR has also been reported to be suppressed in response to morin treatment (Maharjan et al., 2023). Based on these studies, the combination of morin with Dox may be a therapeutic strategy for treating TNBC (Maharjan et al., 2023).

### 4.26 7R-acetylmelodorinol

**FIGURE 2:**
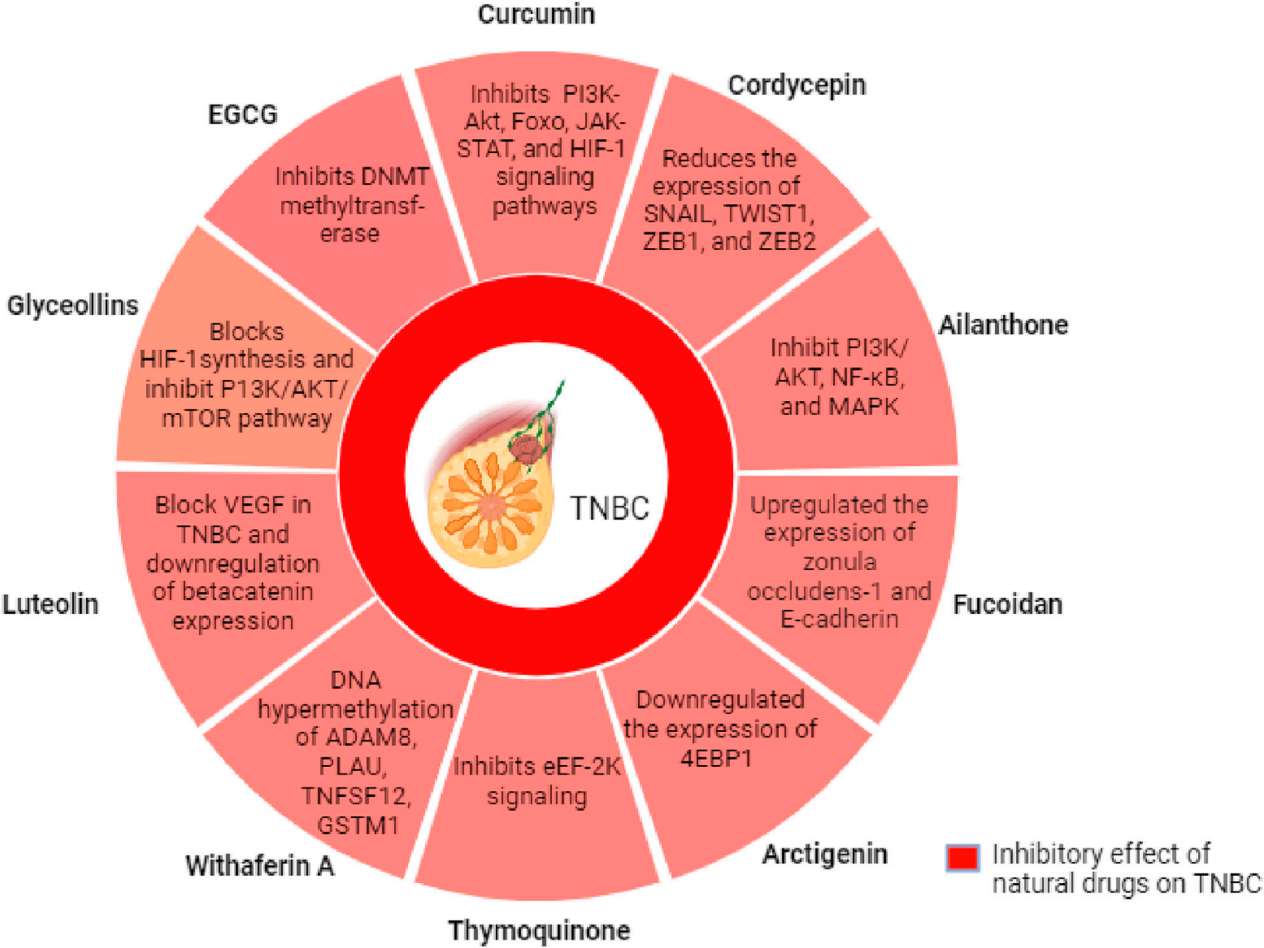
Mechanism of action of selected natural compounds for the suppression of growth and migration of TNBC cells.

7R-acetylmelodorinol (7R-AMDL) is a main compound isolated from the stem bark of *Xylopia Pierrei* (Chokchaisiri et al., 2017). It has been reported to significantly decrease the MDA-MB-231 cell viability, according to MTT and clonogenic experimentation. The treatment of 7R-AMDL (8 µM) leads to substantial activation of the caspase cascade, upregulation of death receptors and their ligands, activation of the intrinsic and extrinsic apoptosis pathways, and a reduction in the expression of Bcl-2-like 1 (BCL2L1/Bcl-xL). In addition, inhibition of cell migration and invasion by 7R-AMDL has been associated with reduced expression of metallopeptidase 9 (MMP-9) and activation of nuclear factor kappa B (NF-κB) in MDA-MB-231 cells (Thepmalee et al., 2023). These studies highlights that 7R-AMDL could be a promising therapeutic option for treating TNBC (Thepmalee et al., 2023). We have summarized this article in Table 1 and Figure 2.

**TABLE 1:** Natural compounds and their possible mechanism of action for the management of TNBC.

Natural drugs	Target	Mechanism	Result	Reference
Epigallocatechin-3-gallate (EGCG)	DNA methyltransferase	Inhibits DNMT and catechol-O-methyltransferase	↓Proliferation of TNBC cells	Schröder et al. (2020)
Thymoquinone	eEF-2K, Src/FAK, and Akt	Inhibits eEF-2K signaling	↓Cell proliferation, migration/invasion, and tumor growth	Kabil et al. (2018)
Curcumin	PTGS2, EP300, STAT3, TNF, GSK3B, PPARG, AKT1, EGFR, NFE2L2, and MMP9	Inhibits EGFR tyrosine kinase, PI3K-Akt, Foxo, JAK-STAT, and HIF-1 signaling pathways	↓Proliferation of MDA-MB-231 cells and induced apoptosis	Deng et al. (2022)
Calycosin	ER-α30	Decrease ER-α30 expression in tumor tissues	Inhibition of TNBC cell growth and metastasis	Li et al. (2023)
*Mangifera indica*	PKM2	PKM2 inhibition	Block growth of MDA-MB231 cells	Rasul et al. (2021)
Cordycepin	—	Reduces the expression of EMT-related transcription factors (SNAIL, SLUG, TWIST1, ZEB1, and ZEB2) in TNBC cells	Decreased migration of TNBC cells	Wei et al. (2022)
Ailanthone	PI3K/Akt, NF-κB, and MAPK	Inhibit PI3K/Akt, NF-κB, and MAPK pathways	Controlled breast cancer metastases	Wang et al. (2023)
Polyphyllin III or dioscin	ACSL4	Induced ferroptosis via ACSL4-mediated lipid peroxidation elevation	Proliferation-inhibitory effect on MDA-MB-231 triple-negative breast cancer cells	Zhou et al. (2021)
Tanshinone IIA	VEGF and mTOR/p70S6K/4E-BP1 signaling pathway	Inhibits HIF-1α and VEGF expression in breast cancer cells via mTOR/p70S6K/RPS6/4E-BP1 signaling pathway	Suppression of growth and angiogenesis of cancer cells	Li et al. (2015)
Glyceollins	P13K/Akt/mTOR pathway	Block HIF-1 synthesis and inhibits P13K/Akt/mTOR pathway	Suppress tumorigenesis in TNBC cell lines	Lee et al. (2015)
Amentoflavone	Hedgehog/Gli1 signaling pathway	Regulates the Hedgehog/Gli1 signaling pathway	Inhibits tumor sphere formation	Bao et al. (2019)
Sulforaphane	Paxillin, IQGAP1, FAK, PAK2, and ROCK	Inhibit MEK and ERK phosphorylation, TGF-β1-induced actin stress fiber formation, and the expression of paxillin, IQGAP1, FAK, PAK2, and ROCK	Inhibit TGF-β1-induced migration and invasion in breast cancer cells	Zhang et al. (2022)
Fisetin	Kinase signaling	Target kinase signaling	Inhibit migration of TNBC cells	Shahi Thakuri et al. (2020)
Ginsenoside 20(S)-protopanaxadiol	RGFR-mediated MAPK pathway	Target the RGFR-mediated MAPK pathway	Inhibit tumor metastasis of TNBC	Peng et al. (2019)
Quercetin	IGF1R and Erk1/2	Inhibits the expression of EMT transcription factors SNAIL and SLUG	Blocks the metastasis of TNBC	Chen et al. (2021)
Luteolin	SGK1-FOXO3a-BNIP3 pathway	Block VEGF in TNBC and Downregulate beta-catenin expression	Inhibits proliferation, mammosphere formation in TNBC, and suppresses the metastasis	Cook, 2018; Wu et al. (2023)
Astragalus polysaccharides	PIK3CG/Akt/BCL2 pathway	Inhibits the PIK3CG/Akt/Bcl2 pathway	Inhibit TNBC cell activity, reduce invasion, and promotes apoptosis	Liu et al. (2019b)
4β-hydroxywithanolide E (4-HW)	P13/Akt signaling pathway	Inhibits P13/Akt signaling pathway	Inhibits the viability of TNBC cells and induced apoptosis/necrosis	Wang et al. (2019b)
Fucoidan	HIF1-α signaling pathway	Downregulate the expression of N-cadherin, and vimentin, and upregulated the expression of zonula occludens-1 and E-cadherin	Suppressinvasion and migration of MDA-MB-231 cells	Li et al. (2019)
Withaferin A	P13/Akt signaling pathway	DNA hypermethylation of tumor-promoting genes. ADAM8, PLAU, TNFSF12, GSTM1, or mitochondrial metabolism	Silences HER2/PR/ESR-dependent gene expression	vel Szic et al. (2017)
Arctigenin	STAT3 and Akt pathway and 4EBP1	Inhibits the Akt pathway by reactivating the PP2A, ↓4EBP1 expression in MDA-MB-231 and BT549 cells	Inhibits the proliferation and epithelial to mesenchymal transition in MDA-MB-231 and BT549 cells	Huang et al. (2017), Luo et al. (2021)
Propolis and EGCG	DNA methyltransferase	Demethylate promoter region of RASSF1A in BT-549 cells	Reduce cell viability of BT-20, BT-549, MDA-MB-231, and MDA-MB-436 cells	Assumpção et al. (2020)
Sanguinarine	Akt/PI3K signaling pathway	↑mRNA expression of BCL211, TNFRSF, and CASP in MDA-MB-468 cells Inhibits Akt expression	↑Apoptosis, cell cycle arrest at S-phase	Messeha et al. (2023)
Eupalinolide J	STAT3 signaling pathway	Promotes degradation of STAT3 in MDA-MB-231 and MDA-MB-468	Suppress the growth of TNBC cells	Lou et al. (2019)
Ursolic acid	p53 signaling pathway	↓MMP-2/9 levels ↓Bcl-2 expression	Cell cycle arrest at G2/M phase ↓Cell proliferation	Zhang et al. (2021c)
Morin	EGFR/STAT3 signaling pathways	Suppress FOXM1 and attenuation of EGFR/STAT3 signaling pathways in MDA-MB-231 TNBC cells	Downregulate the EGFR and STAT3 phosphorylation	Maharjan et al. (2023)
7R-acetylmelodorinol (7R-AMDL)	—	Decrease MMP-9 expression and NF-κβ activation	Decrease viability of MDA-MB-231 TNBC cells	Thepmalee et al. (2023)

↑ - increase in expression, ↓ - decrease in expression.

Given the above-mentioned experimental/laboratory results utilizing cell-based and xenograft models, the scientific community is highly optimistic about coming up with certain natural compounds or their combinations that could work effectively against TNBC in clinics. In addition, the effective utilization of some natural compounds as adjuvants could be a boost to our fight against this deadly and aggressive form of breast cancer. Table 2 enlists some natural compounds and/or their mixture in the different stages of clinical trials for the management of TNBC.

**TABLE 2:** Natural compounds/their combination currently in clinical trials for the management of triple-negative breast cancer.

Compounds	Identifier	Phase of clinical trials	Stages of TNBC	References
Neoadjuvant Talazoparib	NCT03499353	Phase II	Early-stage TNBC	Litton et al. (2023)
Sacituzumab govitecan	NCT02574455	Phase 3	Metastatic TNBC	O’Shaughnessy et al. (2022)
Sacituzumab govitecan + Talazoparib	NCT04039230	Phase II	Metastatic TNBC	Abelman et al. (2022)
Combination of Camrelizumab with Apatinib and Eribulin	NCT04303741	Phase II	Advance TNBC	Liu et al. (2022)
Camrelizumab + Apatinib	NCT03394287	Phase II	Advance TNBC	Liu et al. (2020)
Entinostat with Nivolumab ± Ipilimumab	NCT0245362, ETCTN-9844	Phase 1	Advance TNBC	Roussos Torres et al. (2021)
Atezolizumab + Entinostat	NCT02708680	Phase II	Advance TNBC	O’Shaughnessy et al. (2020)
Neoadjuvant Ipilimumab and Nivolumab combined with paclitaxel	ACTRN12617000651381	Phase II	Stage III TNBC	Loi et al. (2024)

## 5 Conclusion

TNBC continues to remain the most aggressive form of breast cancer with a poor prognosis due to several molecular subtypes and associated genetic modulations. Apart from genetics, epigenetics play a very crucial role in the etiology of TNBC. Epigenetic alterations like DNA methylation and histone modifications modify the gene expression, influence tumorigenesis and metastasis. Newer epigenetic information and knowledge gained through experimental work have resulted in the understanding of some unknown TNBC mechanisms. This article highlights several natural compounds. which have the potential to suppress the growth and metastasis of TNBC cells via interference and modulation of different signaling cascades, such as P13/Akt, PIK3CG/Bcl-2, EGFR/STAT3 and MAPK. However, further studies are required to utilize the potential of natural compounds either in combination or as an adjuvant in clinics.
